# Time to poor treatment outcome and its predictors among drug-resistant tuberculosis patients on second-line anti-tuberculosis treatment in Amhara region, Ethiopia: retrospective cohort study

**DOI:** 10.1186/s12889-019-7838-2

**Published:** 2019-11-08

**Authors:** Daniel Bekele Ketema, Kindie Fentahun Muchie, Asrat Atsedeweyn Andargie

**Affiliations:** 1 Department of Public Health, College of Health Sciences, Debre Markos University, Debre Markos, Ethiopia; 2 Department of Epidemiology and Biostatistics, School of Public Health, College of Medicine and Health Sciences, Bahir Dar University, Bahir Dar, Ethiopia; 3 Department of Epidemiology and Biostatistics, Institute of Public Health, College of Medicine and Health Sciences, University of Gondar, Gondar, Ethiopia

**Keywords:** Poor treatment outcome, Drug resistant tuberculosis, Second line, Weight change

## Abstract

**Background:**

Treatment of drug-resistant tuberculosis is often more complex and toxic with longer treatment time and poor treatment outcomes including treatment failure or death. Monitoring drug-resistant tuberculosis therapy including early identification of prognostic factors and close monitoring of body weight in resource-limited settings is crucial to ensure successful treatment. Therefore, this study was conducted to assess time to poor treatment outcome which is defined as the proportion of all patients who died or failed treatment and its predictors among drug-resistant tuberculosis patients on second-line anti-tuberculosis treatment in Amhara region, Ethiopia.

**Methods:**

A retrospective cohort study was conducted on all patients who started drug-resistant tuberculosis therapy from September 1, 2010 through December 31, 2017, at the University of Gondar Comprehensive Specialized Hospital, Boru-Meda Hospital, and Debre-Markos Referral Hospital in Amhara Region, Ethiopia. Data were entered using Epi-data Version 3.1 and analyzed using R version 3.41 software. The survival time was estimated using Kaplan-Meier survival curve and the survival time between different categorical variables were compared using the log rank test. Event time ratio with 95% confidence interval (CI) and *p*-value less than 0.05 were used to measure the strength of association and to declare statistically significant predictors respectively.

**Results:**

A total of 508 patients with a median age of 28.5 [IQR: 22–40] years were included in this study. The overall cumulative survival probability of patients at the end of 24 months was 79% [95% CI,75,84%]. Rate of body weight change [Adjusted time ratio (ATR) = 5; 95% CI: 3.2, 7.7], secondary and above level of education [ATR = 2.3;95% CI:1.2,2.9], being non-anemic [ATR = 2.8,95% CI:1.2,3.8], being non-diabetic [ATR = 3.4;95% CI:1.3,8.8], without clinical complications [ATR = 7.6;95% CI:4.2,13.9], HIV negative [ATR = 1. 94:95% CI:1.35,2.35] and residing in rural [ATR = 0.51,95% CI:0.30,0.86] were predictors of time to poor treatment outcomes.

**Conclusion:**

The survival rate of tuberculosis patients was higher at end of follow up relative to other studies. However, poor treatment outcome was higher in early phase of therapy. Educational level, rural residence, HIV/AIDS, diabetes mellitus, previous treatment, clinical complication, rate of body weight change and smoking history were significant predictors of time to poor treatment outcome. Therefore, intervention programs should focus on the identified factors to improve survival time of drug-resistant tuberculosis patients.

## Background

Despite the global efforts to control tuberculosis (TB), drug resistant TB (DR-TB) has become an emergent public health problem and causes tremendous morbidity and mortality worldwide, and therefore, it requires treatment with a second-line regimen [1]. According to the World Health Organization (WHO) 2018 global TB report in 2017, 558,000 drug-resistant cases were estimated to be diagnosed globally and 3.5% new TB cases and 18% of previously treated cases had MDR/RR-TB [2].

The incidence of DR-TB is increasing in resource-poor countries [3] where access to sputum culture is limited. Therefore, simple measures of treatment responses like body weight need to be considered. Ethiopia is one of the 30 high burden DR-TB countries identified by WHO. According to the Global TB Report 2018, in Ethiopia, an estimated 2.7% of new cases and 14% previously treated cases developed MDR/RR-TB in 2017 [2].

Given the burden of MDR-TB, the Ethiopian government has initiated a national MDR-TB treatment program with two designed treatment centers: St. Peters Tuberculosis Specialized Hospital and Gondar University Hospital in 2010 [4]. Additional MDR-TB treatment centers are being expanded to the entire country.

Time to poor treatment outcome is affected by numerous sociodemographic, clinical, and behavioral factors. The common sociodemographic factors include: age, gender, level of education and residence [5–11]. The clinical factors include: Human immunodeficiency virus (HIV), Diabetes Mellitus (DM), TB related clinical complication, having previously received anti-TB treatment, anemia and being underweight during the initiation of treatment [7, 9, 12–17]. Additionally, drinking alcohol, smoking and chat chewing were among the common behavioral factors that increase the incidence of poor treatment outcome among tuberculosis patients [5, 18, 19].

Several studies demonstrated the relevance of weight variation to predict DR-TB treatment outcomes [20–24]. However, these studies did not identify the effect of longitudinal weight trajectory on the risks of poor treatment outcome. In this study, by using the advantage of a joint modeling approach we estimated the effects of longitudinal weight trajectory rate on the risk of poor treatment outcome. Due to this statistical modeling difference, this paper clearly aimed to identified weight as indicator of poor treatment outcomes of DR-TB. This finding may be relevant in public health, especially in resource-constrained settings:

Therefore, monitoring weight gain during treatment is a practical tool to identify patients whose treatment is failing and who may benefit from earlier re-evaluation, longer treatment, or more frequent follow-up. Multiple end points measurement collected simultaneously in the DR-TB treatment program provides a valuable oppurtunity to assess predictors of time to poor treatment outcome. However, conventional survival analysis commonly applied in the analysis of time-to-event data may introduce time-dependent bias as the values of weight can vary over time. Further, separate analyses of longitudinal weight change and time to poor treatment outcome data will typically be inappropriate when one is interested in the relationship between the longitudinal outcomes and the time-to-event data and may lead to biased estimates as well as a loss of efficiency. However, a joint modeling approach can overcome these difficulties by simultaneously analyzing the longitudinal outcome variables with the duration process [25–27]. Therefore, this study tried to assess time to poor treatment outcome and its predictors using a joint modeling approach among drug-resistant tuberculosis patients on second-line anti-tuberculosis treatment in Amhara region, Ethiopia.

## Methods

### Study design and area

A facility based retrospective cohort study was carried out from September 1, 2010 over December 31, 2017 among DR-TB patients receiving second-line anti-tuberculosis treatment in selected hospitals in Amhara region, Ethiopia. The hospitals that were included in this study were University of Gondar Comprehensive Specialized Hospital, Boru-Meda Hospital, and Debre-Markos Referral Hospital. These hospitals represent for 80% of DR-TB patients on second-line anti-tuberculosis who receive service in Amhara region [28].

University of Gondar Comprehensive Specialized Hospital, which is located in North Gondar Administrative Zone, started DR-TB treatment as a pilot program with the Global Health Commute (GHC) to treat patients. The hospital started service as a national response to the emerging threat of DR-TB on September 2010 [28]. The second study site was Boru-Meda Hospital, located 10 km from Dessie, the capital of South Wello, and 441 km from Addis Ababa, capital of Ethiopia. The treatment initiating center was established in 2013 and has served more than 174 DR-TB patients until December 31, 2017. The third setting was Debre-Markos Referral Hospital, the capital of East Gojjam administrative Zone. It is 297 km from Addis Ababa and 264 km from Bahir-Dar, the capital of the region [28].

### Study population

All drug-resistant TB patients who were culture positive at the start of treatment and who had at least one body weight record in the follow up period were included in the study. Patients who had incomplete data on the outcome variables and pregnant women were excluded from the study.

### Sample size determination

All DR-TB patients who were enrolled at the University of Gondar’s Comprehensive Specialized Hospital, Debre Markos Referral Hospital and Boru-Meda Hospital from September 1, 2010 through December 31, 2017 were included in this study. The minimum adequacy of samples was determined using STATA 14.1 software based on sample size determination formula of survival analysis for single arm follow up study. Accordingly, a total of 508 study participants were included in this study.

### Variables of the study

The dependent variable of this study was time to poor treatment outcome described using month. The independent variables were socio-demographic variables like age at baseline, marital status, residence, educational status, sex, occupational status, and religion. Behavioral variables included in this study were smoking and alcohol use. Clinical variables include type of resistant (RR resistance, MDR, poly resistance, XDR), clinical complication, baseline smear result, comorbidities, anemia, previous history of TB, episodes of previous TB treatment, and length of hospital stay. Additionally, time-varying endogenous covariate like body weight recorded in kilograms (kg) from treatment start (baseline) and repeatedly measured on a monthly basis were the independent variables.

### Operational definitions

DR-TB treatment outcome for this study was defined based on WHO definitions and reporting framework for TB guidelines as cured, treatment completed, treatment failed, died, lost to follow-up and not evaluated [29].

Treatment failure was defined as treatment terminated or a need for permanent regimen change of at least two anti-TB drugs because of lack of conversion by the end of the intensive phase, or bacteriological reversion in the continuation phase after conversion to negative after intensive phase, or evidence of additional acquired resistant to fluoroquinolones or second-line injectable drugs, or adverse drug reactions [30] .

Death: referred to death for any reason during the course of treatment.

Follow-up: defined as the time from commencement of treatment up to any treatment outcome observed.

Poor treatment outcome: is the proportion of all patients who died or failed treatment.

Time to poor treatment outcome: is the time gap in months between the beginning of DR-TB treatment to the date of death or treatment failure.

Censored: patients were censored when their TB treatment outcome was labeled as cured, completed, transferred out, lost to follow-up or were still on treatment at the end of the study.

Anemia: patients were considered as anemic if their Hemoglobin level is < 12 g/dl for female and children and less than 13 g/dl for men [31].

Event Time Ratio: > 1 means factor accelerates survival time, or leads to longer survival.

Event Time Ratio: < 1 means factor decelerate the survival time, or leads to shorter survival.

Current value of body weight: Body weight measurement at particular time point.

Assoct (association between body weight the risk of poor treatment outcome) (refer Table 3**):** Quantifies the effect of the underlying longitudinal body weight to the risk for poor treatment outcome.

### Treatment regimens

In Ethiopia, DR-TB treatment is given based on the recommendations from the 2018 WHO treatment guidelines [32]. All newly diagnosed DR-TB patients receive a standardized regimen of first- and second-line anti-TB drugs. The treatment regimen consists of an 8-month intensive phase with a combination of pyrazinamide (Z), capreomycin (CM), levofloxacin (Lfx) and prothionamide (Pto) or ethionamide (Eto) and cycloserine (Cs), a 12-month continuation phase with a combination of pyrazinamide (Z), levofloxacin (Lfx), prothionamide (Pto) or ethionamide (Eto), and cycloserine (Cs) [31].

### Data collection tool and procedure

This study used secondary data that were collected using a structured data extraction checklist developed based on the national DR-TB treatment guideline and registration book. Data were extracted from patients’ MDR-TB registration books and medical records. The registration book contained a number of variables including socio-demographic characteristics (age, sex, residence, marital status, educational status, occupation, religion, and treatment supporter), clinical variables (HIV status and other comorbidities, site of TB disease, number of previous TB treatments, initial MDR-TB regimen, initial regimen change, initial sputum and culture result, adverse drug effects, height and baseline weight and monthly weight record across all follow-up time), and laboratory profile (haemoglobin (Hgb)). Data were collected by three trained healthcare workers who were working in the MDR-TB treatment centre.

### Data quality management

Training was given to data collectors and supervisors for 2 days before the data collection period. The training focused on the objective of the study and how to retrieve records as per the data extraction sheet. The data extraction sheets were pre-tested for consistency, understanding of the tool, and completeness of data for charts. Necessary adjustments were made on the final data collection sheet after the pretest. During the data collection period, random samples were extracted to cross-check with the actual patient data.

### Data quality management

Training on the objective of the study and how to retrieve records as per the data extraction sheet was given to data collectors and supervisors for 2 days before data collection. The data extraction sheets were pre-tested for consistency, understanding of the tool, and completeness of data for charts. Necessary adjustments for the final data collection sheet were made after the pretest. During the data collection period, random samples were extracted to cross-check with the actual patient data.

### Data processing and analysis

Collected data were checked for inconsistencies, coding error, completeness, accuracy, clarity, and missing values before data entry. The data were entered using EPI-info version 7 and then were exported to the R V.3.4.3 statistical software for further analysis.

Descriptive measures such as means, median, IQR, percentages, frequencies, standard deviations (SD), and graphing were used to characterize the study participants. Time for poor treatment outcome was estimated using the Kaplan-Meier (KM) method. The log-rank test was used to compare between groups of baseline categorical variables. A more parsimonious model was chosen by means of the likelihood ratio test, Akaike information criteria (AIC) and graphical methods. Weibull Accelerated failure was parsimonious for the survival model. The factors significantly associated with poor treatment outcome in the univariable models at *p* values of less than 0.2 were included in the multivariable survival model and further examined in the joint model. Time ratio (TR) with 95% CI was used to select variables which have a significant association with poor treatment outcome in the joint model. A significance level of 0.05 was taken as a cut point for all statistical tests. To estimate the effects of longitudinal weight change to the risks of poor treatment outcome, the study constructed the complete true history of weight for each study participant using the linear mixed model by considering effects of baseline covariate on body weight evolution. Finally, Weibull Accelerated failure with Gauss-Hermit and linear mixed model with random intercept and random slope fitted jointly using R package JM with the current value and slope parameterization.

## Results

### Demographic characteristics

From September 2010 through December 2017, a total of 572 patients initiated DR-TB treatment in the selected hospitals. Of these, 508 patients were eligible and were included in the analysis. The median age of study participants was 28.50 [IQR: 22–40 years], and 55.70% were male (Table 1). The median baseline BMI was 16.43 [IQR: 14.82–18.00] Kg/m ^2^. More than two third (79.53%) of study participants had been previously treated for TB, with a median of 2 prior treatments. The median length of hospital stay of patients was 66.50 [IQR: 35.50–120] days for those who had experienced poor treatment outcome and 52.00 [IQR: 30–90] days for those who did not.

**Table 1:** Baseline socio-demographic characteristics of DR-TB patients on second line anti-TB treatment in Amhara region, Ethiopia (2010 to 2017)

Variables	Frequency	Percent
Sex		
Male	283	55.7
Female	225	44.3
Age		
< = 24	165	32.5
25–44	258	50.8
≥ 45	85	16.7
Marital status(*n* = 494)		
Never married	248	50.2
Married	183	37.1
Divorced	47	9.5
Widowed	16	3.2
Education		
No education	227	44.7
Primary	142	28.0
Secondary and above	139	27.3
Occupation		
Farmer	145	28.5
House wife	87	17.1
Daily Laborer	66	13.0
Government employed	112	22.0
Student	98	19.4
Religion		
Orthodox Christian	414	81.5
Muslim	86	16.9
Others*	8	1.6
Residence		
Urban	243	47.8
Rural	265	52.2

Other * = Catholic and protestant

### Clinical characteristics of study participants

The majority of the patients (88%) were diagnosed with pulmonary TB. During initiation of treatment nearly 78.5% were sputum smear positive. Additionally, 123 patients (24.21%) were HIV infected. (Table 2).

**Table 2:** Baseline clinical characteristics of DR-TB patients on second line anti-TB treatment in Amhara region, Ethiopia

Variables	Frequency	Percent
Sputum smear		
Positive	400	78.5
Negative	108	21.5
BMI		
< 18.5	388	76.4
≥ 18.5	120	23.6
Co-existing diabetes		
Yes	16	3.1
No	492	96.9
CKD		
Yes	7	1.4
No	501	98.6
HIV		
Positive	123	24.2
Negative	385	75.8
COPD		
Yes	88	17.32
No	420	82.68
Hemoglobin status		
Anemic	392	77.2
Non anemic	116	22.8
HTN		
Yes	15	2.9
No	493	97.1
Previous TB treatment		
Yes	404	79.5
No	104	20.5
Resistant type		
R or H	288	56.7
MDR	220	43.3
TB type		
Pulmonary	447	88.0
Disseminate	61	12.0
Cigarette smoker		
Yes	72	14.2
No	436	85.8

*COPD* Chronic obstructed pulmonary disease, *BMI* body mass index, *HTN* hypertension, *HIV* human immune virus, *CKD* chronic kidney disease = refimpsine, *H* isoniazid, *MDR* multi drug resistant

### Survival probability of DR-TB patients

A total of 508 DR-TB patients were followed for different periods; a minimum of 1 month and a maximum of 24 months with median follow up period of 20 (IQR:18–21) months which.

contributed a total of 8044 person-months observation. The cumulative survival probability of patients at the end of 8 months was 87%, at the end of 12 months was 84%, and at the end of 24 months was 79%. The median survival time for this study could not be determined due to presence of mass censored, instead, the restricted mean survival was 19.26 [95% CI: 18.75, 19.82] months. The overall incidence rate of poor treatment outcome was 10.6 (95% CI: 8.58, 13.11) per 1000 person-months and it was high, 12.3 (95% CI: 10.21, 15,30) person-months, during the first 8 months after second-line anti-TB treatment initiation.

**Fig. 1:**
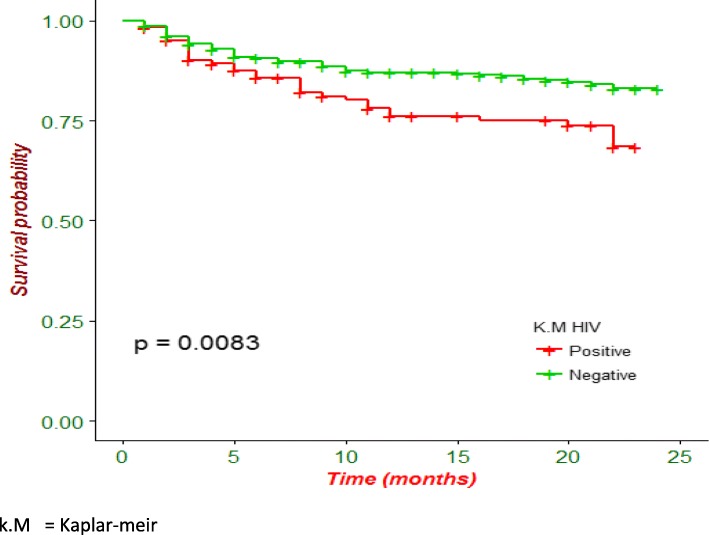
Kaplan-Meier survival curve of drug resistant tuberculosis patients on anti-TB treatment by HIV status in Amhara region, Ethiopia

**Fig. 2:**
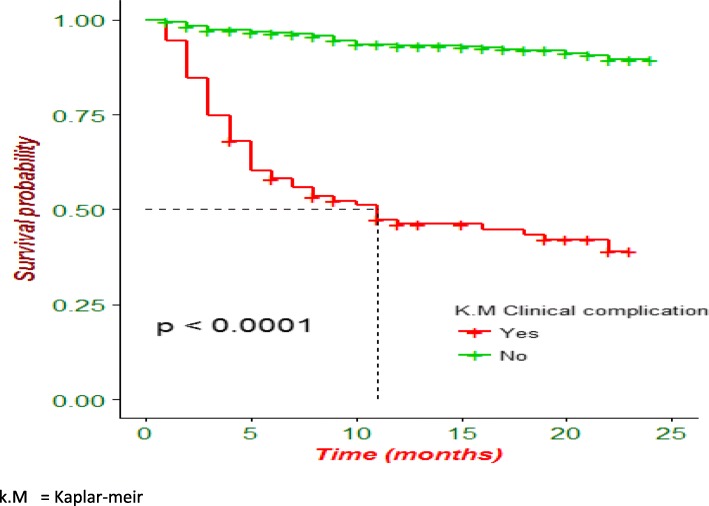
Kaplan-Meier survival curve of drug resistant tuberculosis patients on anti-TB treatment by a clinical complication in Amhara region, Ethiopia

**Fig. 3:**
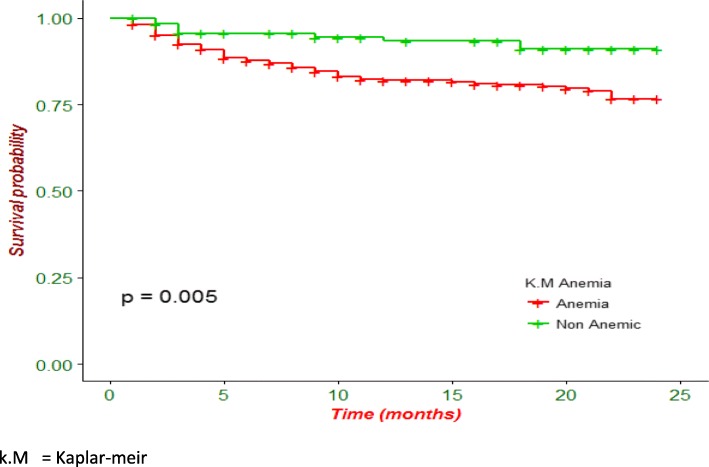
Kaplan-Meier survival curve of drug resistant tuberculosis patients on second-line anti-TB treatment by anemia in Amhara region, Ethiopia

Kaplan-Meier analysis revealed that poor treatment outcome was higher in patients who were HIV-infected (Fig. 1) and in patients presented with any clinical complication (Fig. 2). Additionally, patients with anemia had lower survival probability than their counterparts (Fig. 3).

### Predictors of time to poor treatment outcome

After fitting separate models of the longitudinal process and the survival process, the best-fitted model was chosen and fitted by the joint model. It is assumed that the risk for poor treatment outcome at a particular time point depends on the true level of the body weight at the same time point, but it is not realistic to expect that this parameterization will always be the most appropriate in expressing the correct relationship between the change in body weight and time to poor treatment. To overcome these, both the current value and the slopes of the body weight trajectory’s Parameterizations were used at Weibull-accelerated Failure time.

The study observed strong evidence that both the current value and the slope of weight change of unobserved true body weight is associated with the survival of patients. A unit increase in the current value of body weight showed a 4% [95% CI: 1.2%; 6%], accelerated survival time of patients taking the assumption that the effect of the true body weight is the same in all subgroups of the target population. Whereas, a unit increase in the rate of body weight trajectory accelerated survival time of patients by 5 [95% CI: 3.2; 7.7] times, provided that the true unobserved value remains constant (Table 3). The baseline predictors of time to poor treatment outcome were stated under Table 4.

**Table 3:** Association between longitudinal body weight change and time to poor treatment outcome among DR-TB patients on second line anti TB treatment in Amhara region, Ethiopia

Associate parameter	ATR [95% CI]
Assoct (Current value)	1.04 [1.01 1.06]
Assoct.s (slope (Rate of change))	5.00 [3.20 7.71]

The parameter labeled ‘Assoct’ measures the association between body weight change and the risk for poor treatment outcome

**Table 4:** Survival submodel with slope parameterizations under Weibull accelerated failure time for DR-TB patients in Amhara region, Ethiopia

Variables	Status			
	Event (%)	Censored (%)	CTR[95% CI]	ATR[95% CI]
Age				
< =24	16 (9.7)	149 (90.3)	1.00	1.00
25–44	45 (17.4)	213 (82.6)	0.42 [0.21 0.88]	0.45 [0.14 1.84]
45 and above	25 (29.4)	60 (70.6)	0.18 [0.17 0.43]	0.28 [0.10 0.68]
Residence				
Urban	27 (11.1)	216 (88.9)	1.00	1.00
Rural	59 (22.3)	206 (77.7)	0.36 [0.20 0.67]	0.51 [0.30 0.86]
TB complication				
Yes	52 (57.1)	39 (42.9)	1.00	1.00
No	34 (8.2)	383 (91.8)	15 [7.59 29.72]	7.65 [4.22 13.88]
Sputum smear				
Positive	72 (18.0)	328 (82.0)	1.00	1.00
Negative	14 (13.0)	94 (87.0)	1.56 [0.75 3.27]	1.23 [0.65 2.33]
Education				
No education	50 (22.0)	177 (78.0)	1.00	1.00
Primary	24 (16.9)	118 (83.1)	1.47 [0.79 2.73]	0.75 [0.41 1.36]
Secondary and above	12 (8.7)	127 (91.3)	3.87 [1.68 8.90]	2.34 [1.34 2.88]
Anemia				
Anemic	75 (24.1)	236 (75.9)	1.00	1.00
Not anemic	11 (5.6)	186 (94.4)	3.37 [1.35 8.41]	2.58 [1.21 3.80]
HIV				
Positive	30 (24.4)	93 (75.6)	1.00	1.00
Negative	56 (14.6)	329 (85.4)	2.15 [1.21 3.84]	1.94 [1.35 2.35]
Diabetes				
Yes	7 (43.7)	9 (56.3)	1.00	1.00
No	79 (16.1)	413 (83.9)	4.52 [1.64 12.46]	3.45 [1.34 8.86]
TB treatment				
No treatment	9 (12.2)	65 (87.8)	1.00	1.00
One and above	77 (17.7)	370 (82.3)	0.57 [0.22 1.49]	0.37 [0.16 0.86]
Smoking history				
Yes	18 (25.7)	52 (74.23	1.00	1.00
No	68 (15.5)	370 (84.5)	2.05 [1.04 4.02]	1.29 [1.07 2.88]

*CTR* Crude time ratio, *ATR* Adjusted time ratio, *TB* tuberculosis, TB complication (pneumonia, hemoptysis, pneumothorax, Corpulmonale

## Discussion

This study aimed to estimate the time to poor treatment outcome of patients and its predictors as well as effects of body weight trajectory rate on poor treatment outcome of DR-TB patients in Amhara region.

The other all cumulative survival probability of patients at the end of treatment was 79% [95% CI: 75, 84%], which is in agreement with a research conducted in St Peter’s specialized TB Hospital, Addis Ababa, northest Ethiopia and China [15, 30, 33, 34], but higher than the WHO target of at least 75% treatment success of DR-TB. This promising survival probability of the patients in the Amhara region may be due to some reasons related to the program. All patients in the study area received economic assistant for transport, additional food, and house rent if needed throughout therapy. Furthermore, all patients were screened for any comorbidity and clinical complications upon enrolment and were offered appropriate therapy for the problems.

In this study, there was a significant difference in the survival of TB patients with Diabetes Mellitus (DM) and without DM. Patients without DM had 3.45 fold longer survival time than diabetic patients. This association has been demonstrated in previous studies [9, 11, 35]. A possible explanation might be that patients with diabetes mellitus have impaired immunity compared to healthy individuals, and sequalae of diabetes may potentiate the adverse effects of anti-TB drugs.

This study also found HIV infection as an important predictor of poor treatment outcome during the course of therapy among DR-TB patients. Regardless of CD4 cell count, patients without HIV survived 1.94 times higher than patients who had HIV infection. TB/HIV co-infected patients had a substantial probability for experiencing poor treatment outcomes which is consistent with the results of other studies [9, 12]. This might be due to the fact that DR-TB with HIV co-infection faces lots of challenges which include:- high pill burden and adverse drug reactions that result in a high incidence of adverse effects [31].

Additionally, this study also demonstrated that anemia substantially increases the risk of experiencing death or treatment failure compared to non-anemic patients during anti-TB treatment. Recent studies have found similar findings where anemia is associated with poor treatment outcomes [16, 17, 30]. In this study, patients who were non-anemic were 2.80 times more likely to survive compared to anemic patients. This might be related to higher adverse drug effects among anemic patients during anti-TB treatment. In addition, anemic patients might have increased risks of infection and may have a compromised immunity which contributes to the advancement of disease progression, risk of death, and treatment failure during anti TB treatment. Moreover, nearly 80% of anemic patients were found to be underweight at baseline in the current study.

Then number of previous episodes of TB was also associated with the time to poor treatment outcomes in this study. Patients who had one or more episodes of anti-tuberculosis treatment might create greater antibiotic resistant with the subsequent MDR-TB [9, 14]. Previous tuberculosis treatment status is established as a risk factor for drug-resistant TB in different scientific studies [36–38]. This might be patients with previous TB treatment are difficult to manage and might be infectious for a longer period of time, and this longer exposure to anti-tuberculosis drugs causes toxicity and greater incidence of adverse effects. But the result was contradictory other research conducted in South Korea, which revealed no treatment outcome differences between patients with or without previous TB treatment history [13]. This difference might be due to the difference in the living conditions of patients and access to medical services.

In this study tuberculosis-related clinical complications during the course of treatment were associated with poor treatment outcomes. The presence of clinical complications due to pneumonia, pneumothorax, hemoptysis, and cor pulmonale in the course of anti-tuberculosis treatment may indicate the advancement of diseases and subsequent increase in the risks of experiencing poor treatment outcomes. This finding was in agreement with studies conducted in Ethiopia, which showed that patients with clinical complications had a high risks of death compared to those without clinical complications [5, 33, 34].

Additionally, poor treatment outcome was associated with place of residence. Urban residents had a longer survival time than rural residents. This might be due to better access of urban residents to treatment service and the level of the awareness with regards to the disease.

In the present study, as in several previous ones [5, 18] an association of poor DR-TB treatment outcomes with regard to cigarette smoking was found. Cigarette smoking indirectly affects the DR-TB prognosis through manipulating other factors including suppression of anti TB drug activity.

This study also showed that both unobserved current value of weight and rates of weight change were associated with poor treatment outcome. Weight variation during tuberculosis therapy follow up can predict treatment outcome [20]. Patients losing weight in the course of their treatment had a higher risk for poor treatment outcomes. In this study, patients who were on the censored category gained weight by the rate of 0.47 kg, whereas body weight decreased over time by the rate 0.10 kg for those who had experienced poor treatment outcomes.

Several studies demonstrated the relevance of weight variation to predict DR-TB treatment outcomes [20–24]. However, these studies didn’t identify the effect of longitudinal weight trajectory on the risks of poor treatment outcome. In this study, by using a joint modeling approach we estimated the effects of longitudinal weight trajectory rate on the survival of the patients. Due to this statistical modeling difference, this paper clearly identified weight as an important indicators of TB treatment outcome. This finding may be relevant in public health, especially in resource-constrained settings: - weight can be used as predictor of TB outcome and how patients progress during treatment. Moreover, changes in weight could be observable during therapy.

This study had some limitations. Since the study was based on secondary data, potentially important variable like socio-economic status, radiological findings, nutritional variables, CD4 count, which assumed to have an association with poor treatment outcome were not collected. Non-TB related deaths such as accidents or other chronic diseases may cause death in the course of treatment, but the specific causes of death were not available.

## Conclusion

This study conclude that survival rate of patients was higher at end of follow up. However, poor treatment outcome was high in early phase of therapy. Educational level, residence, HIV/ADIS, diabetes mellitus, previous treatment, clinical complication and smoking history were significant predictors of time to poor treatment outcome. Moreover, weight was identified as a predictor of treatment outcome. Patients with weight loss should be followed more closely, as they are at greater risk for poor treatment outcome. Improving targeted social assistance to TB patients who are not educated and rural residents, investigating all possible cases of past TB episodes; diabetes and HIV screening and active control should be incorporated into the follow-up evaluation. Close follow up to prevent the occurrence of early clinical complications, and close monitoring of patients diagnosed with anemia should be a priority.

## Data Availability

The datasets used and/or analysed during the current study are available from the corresponding author on reasonable request.

## Abbreviations

AFT: Accelerated failure time
AIC: Akaike Information Criteria
BMI: Body Mass Index
CKD: Chronic kidney disease
DM: Diabetes Mellitus
DR-TB: Drug-Resistant
GH: Gauss-Hermit
GHC: Global Health Committee
Hgb: Hemoglobin
HIV: Human Immunodeficiency Virus
HTN: Hypertension
IQR: Inter Quartile Range
LMM: Linear Mixed Effect Model
MDR-TB: Multi-Drug Resistant Tuberculosis
RR-TB: Rifampicin Resistant Tuberculosis
TB: Tuberculosis
TIC: Treatment Initiating Center
WHO: World Health Organization, K.M: Kaplar-Meir
